# Exploiting Polyphenol-Mediated Redox Reorientation in Cancer Therapy

**DOI:** 10.3390/ph15121540

**Published:** 2022-12-12

**Authors:** Lei Li, Ping Jin, Yueyue Guan, Maochao Luo, Yu Wang, Bo He, Bowen Li, Kai He, Jiangjun Cao, Canhua Huang, Jingquan Li, Zhisen Shen

**Affiliations:** 1 School of Basic Medical Sciences, State Key Laboratory of Southwestern Chinese Medicine Resources, Chengdu University of Traditional Chinese Medicine, Chengdu 611137, China; 2 State Key Laboratory of Biotherapy and Cancer Center, West China School of Basic Medical Sciences & Forensic Medicine, West China Hospital, Sichuan University, Collaborative Innovation Center for Biotherapy, Chengdu 610041, China; 3 Department of Encephalopathy, Chongqing Hospital of Traditional Chinese Medicine, Chongqing 400021, China; 4 Hainan Cancer Center and Tumor Institute, The First Affiliated Hospital of Hainan Medical University, Haikou 570102, China; 5 Department of Otorhinolaryngology and Head and Neck Surgery, The Affiliated Lihuili Hospital, Ningbo University, Ningbo 315040, China

**Keywords:** polyphenol, oxidative stress, ROS, cancer therapy, drug delivery

## Abstract

Polyphenol, one of the major components that exert the therapeutic effect of Chinese herbal medicine (CHM), comprises several categories, including flavonoids, phenolic acids, lignans and stilbenes, and has long been studied in oncology due to its significant efficacy against cancers in vitro and in vivo. Recent evidence has linked this antitumor activity to the role of polyphenols in the modulation of redox homeostasis (e.g., pro/antioxidative effect) in cancer cells. Dysregulation of redox homeostasis could lead to the overproduction of reactive oxygen species (ROS), resulting in oxidative stress, which is essential for many aspects of tumors, such as tumorigenesis, progression, and drug resistance. Thus, investigating the ROS-mediated anticancer properties of polyphenols is beneficial for the discovery and development of novel pharmacologic agents. In this review, we summarized these extensively studied polyphenols and discussed the regulatory mechanisms related to the modulation of redox homeostasis that are involved in their antitumor property. In addition, we discussed novel technologies and strategies that could promote the development of CHM-derived polyphenols to improve their versatile anticancer properties, including the development of novel delivery systems, chemical modification, and combination with other agents.

## 1. Introduction

Polyphenols, the most common natural antioxidants extracted from plants, primarily function as secondary metabolites to protect plants against reactive oxygen species (ROS), ultraviolet radiation, mechanical damage, pathogens, parasites, and predators [1,2]. It commonly exists in a wide variety of dietary sources, including vegetables, fruits, spices, and natural beverages (e.g., tea, coffee, and wine). Notably, natural herbal medicine [3,4], especially Chinese herbal medicine (CHM), shares the holistic principles of “homology of medicine and food” in traditional Chinese medicine (TCM), which is rich in polyphenols [5,6]. A plethora of studies have identified the numerous activities of polyphenols, including anti-inflammatory, antioxidant, antiviral, and anti-aging activities; therefore, they have been applied and proven efficient in various disease models, including inflammatory bowel disease, coronary heart disease, coronavirus disease 2019, Alzheimer’s disease, and particularly, cancer [7,8,9,10,11].

**Figure 1:**
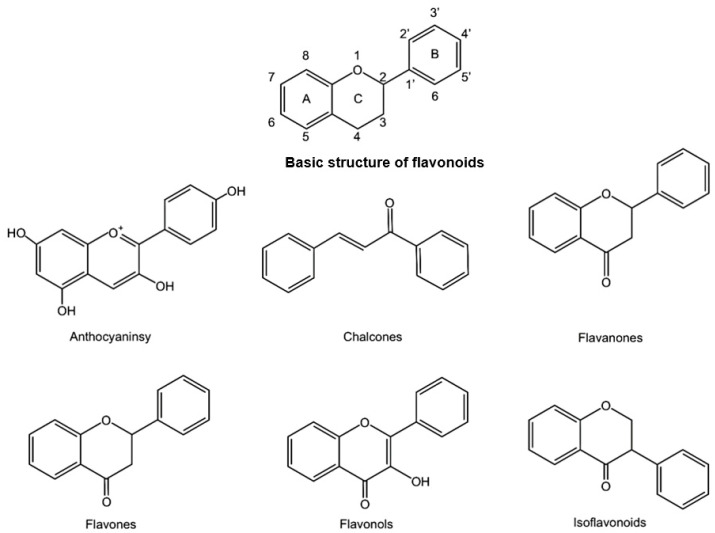
The basic structure of flavonoids and their six main subgroups. This figure was created using Kingdraw.

Polyphenols can be classified into four major categories, including flavonoids, phenolic acids, lignans, and stilbenes, according to their chemical structure [12]. Flavonoids are characterized by a C6-C3-C6 carbon backbone, which is structured as a phenyl ring (ring A) fused with a pyran ring (ring C) that carries another phenyl ring (ring B) substitution (ring C) [13]. Based on this structure, flavonoids are subdivided into six major subclasses: flavonols, flavanols, anthocyanins, flavones, flavanones, and isoflavones [14,15] (Figure 1). Flavonols are ketones with a hydroxyl group at position 3 of ring C [16], while flavanols lack a ketone found in flavonols, instead of a hydroxyl group at position 3 of ring C [17]. Unlike flavonols, anthocyanidins lack a ketone group but contain positively charged oxygen at position 4 of ring C [18]. Phenolic acids, as one of the main classes of phenolic compounds, are produced through the phenylpropanoid pathway and possess phenol moieties and resonance-stabilized structures [19,20]. Phenolic acids are typically bound with esters, glycosides, or amides, but rarely exist in their free form [21]. They mainly consist of two subgroups: hydroxycinnamic and hydroxybenzoic acids [22,23]. Hydroxycinnamic acids are present in simple esters with quinic acid or glucose, containing a saturated tail followed by carboxylic acid [24,25]. Hydroxybenzoic acids, such as vanillic, gallic, protocatechuic, ellagic, and syringic acids, lack tail saturation [17]. Lignans are natural compounds polymerized by phenylpropanoid (C6-C3) derivatives in different ways, usually forming dimers [26] with a few trimers and tetramers [27,28]. The phenylpropane dimers are also divided into lignans or neolignans based on the presence or absence of the 8,8′-bond between phenylpropanoid monomers [26]. Stilbenes contain two benzene rings joined by a molecule of ethanol or ethylene to present a C6-C2-C6 structure [29,30,31]. They are characterized by the presence of a 1,2-diphenylethylene nucleus with hydroxyl groups substituted on the aromatic rings [32], in which resveratrol is well known for its versatile activities [33].

**Figure 2:**
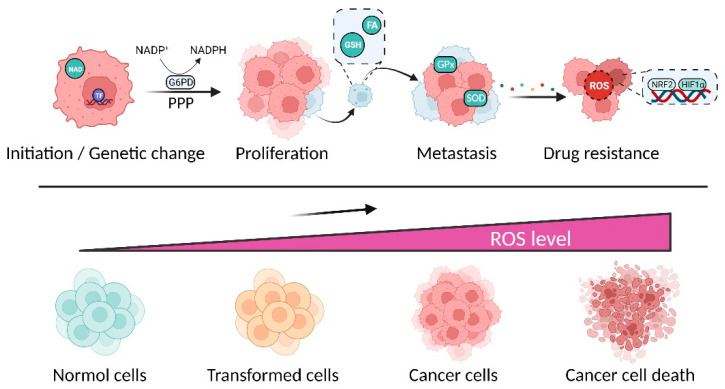
Redox homeostasis involved in cancer evolution. Reactive oxygen species (ROS) almost participate in all aspects of cancer, including initiation, proliferation, metastasis, and drug resistance. Cancer cells evolved a set of robust antioxidant system, including reducing power (e.g., NADPH), non-enzymatic and enzymatic antioxidants, and transcription factors, for adapting to oxidative stress.

ROS contradictorily influence cancer evolution, either triggering tumorigenesis, promoting cancer cell transformation, or leading to tumor regression and even cell death [34]. Intracellular oxidative stress can lead to ROS elevation. To accommodate abnormal ROS levels, cancer cells modify different antioxidant systems, including antioxidant transcription factors, nicotinamide adenine dinucleotide phosphate (NADPH) generation, and metabolic reprogramming, to neutralize oxidative stress [35,36]. During their initiation, stimulating antioxidant transcription factors and increasing NADPH generation enhance the survival of cancer cells [37,38]. Cancer cells are able to activate the pentose phosphate pathway to increase the nucleic acid supply to maintain proliferative capacity. Furthermore, the activation of AMPK signaling and elevation in reductive glutathione and folate metabolism lead to an increase in NADPH synthesis, which protects cancer cells from oxidative stress during metastasis [39]. Moreover, activated transcription factors such as nuclear factor erythroid 2-related factor 2 (NRF2) and hypoxia inducible factor-1α (HIF-1α) promote cancer cells to adapt to drug-mediated oxidative stress (chemoresistance) [40,41]. Therefore, cancer cells exhibit aberrant redox homeostasis to survive the harsh tumor microenvironment (Figure 2).

Disrupting the homeostasis of oxidative stress and a reduced state have been proven to be significant strategies for cancer treatment [42,43]. Some chemotherapeutic agents, such as doxorubicin, oxaliplatin, and paclitaxel, have been shown to exert antitumor effects by disturbing intracellular redox homeostasis [44,45,46], but the side-effect, frequent occurrence of drug resistance and tumor recurrence impaired their use in clinical application. Currently, many natural products, such as natural vitamins, alkaloids, saponins, polypeptides, polysaccharides, and polyphenols, have been employed as antioxidants/prooxidants to treat cancer [47,48,49]. Among them, polyphenols have attracted considerable attention in cancer therapy due to their inherent characteristic of modulating oxidative stress, the critical player in cancer incidence and progression [14,50,51]. Even with the limitations of polyphenols in delivery, targeting, and preservation [52,53,54,55], an increasing amount of evidence has demonstrated that these viable anticancer candidates can regulate redox homeostasis in cancer cells by acting as antioxidants or pro-oxidants to prevent cancer [56,57]. Besides, the overwhelming advantages of polyphenols, including specificity of the response, negligible toxicity, and omnipresence, make them excellent antitumor agents [58]. Currently, researchers are devoted to developing new techniques and strategies for improving the application of polyphenols in cancer treatment [59,60].

In this review, we summarize the therapeutic mechanisms underlying the polyphenol-mediated modulation of redox homeostasis in cancer treatment and drug resistance surmounting. In addition, we discuss novel technologies or methods for optimizing the delivery, targeting, and preservation of polyphenols for effective cancer treatment.

## 2. Polyphenols Modulate Redox Homeostasis for Cancer Therapy

In cancer cells, moderate ROS (the ROS level that is beneficial for tissue turnover and cell proliferation) function as second messengers in cellular physiological processes to modulate cellular signaling and biological reactions to maintain endogenous homeostasis [61], thereby providing advantages for carcinogenesis, metastasis, and cell survival. However, excessive ROS beyond the toxic threshold (the maximum level suitable for cellular homeostasis, triggering the redox homeostasis to activate cell death) could impede tumor progression and trigger cell senescence, apoptosis, or ferroptosis [62,63,64,65]. Therefore, the regulation of cellular redox homeostasis via antioxidants or prooxidants holds significance for cancer therapy. In recent years, modulation of redox homeostasis using natural antioxidants/pro-oxidants for cancer treatment has been largely explored in preclinical research and clinical evaluations [66,67,68,69], and many clinical trials have been implemented (NCT01912820, NCT03493997, NCT00256334, NCT00433576, NCT01717066). As the major sources of natural antioxidants, polyphenols have attracted considerable interest as novel anticancer agents with the dual functions of regulating oxidative stress according to their properties and to the dosage in different tumor models [70,71], including colorectal cancer, breast cancer, lung cancer, gastric cancer, cervical cancer, etc. [72,73,74,75,76]. In this section, we will review the polyphenols that have been reported for their use in cancer therapy, with an emphasis on the underlying molecular mechanisms related to the modulation of redox homeostasis.

### 2.1. Polyphenols Mediate Antioxidant Effects in Cancer Therapy

A variety of polyphenols, including kaempferol, resveratrol, catechins, curcumin, wogonin, quercetin, etc., have been reported to suppress tumors by suspending oxidative stress via various mechanisms. Primarily, the hydroxyl group on polyphenols undergoes hydrogen atom transfer or single electron transfer reactions, thereby scavenging radicals such as hydroxyl, peroxyl, or peroxynitrite [77]. In addition, polyphenols can inhibit the activity of oxidases, including superoxide-producing enzymes, e.g., cyclooxygenase 2 (COX-2) and NADPH oxidase (NOX), or promote the activity of antioxidant enzymes, e.g., superoxide dismutase (SOD), GSH peroxidases (GPXs), peroxiredoxins (PRDXs), and catalase (CAT) [78,79,80,81,82], thus eliminating excessive ROS levels and recovering redox homeostasis. Additionally, polyphenols have been reported to activate classical antioxidant signaling pathways, such as NRF2 and forehead box class O (FOXO), and promote the transcription of antioxidant proteins, such as Skinhead-1, Heme Oxygenase-1 (HO-1), PRDX-2, SOD3, and Glutathione S-transferase-4 (GST-4) [83,84,85,86,87].

#### 2.1.1. Kaempferol

Kaempferol, a well-known natural polyphenol derived from CHMs (e.g., *Tetrastigma hemsleyanum* Diels et Gilg, *Sparganii Rhizoma*, Lysimachiae Herba (dried whole part of *Lysimachia christinae* Hance)) [88,89,90]), has obvious anticancer efficiency in multiple cancer cells [91,92,93]. Kaempferol can promote the expression of NOQ1, SOD1, and HO-1 in HL-60 cells, a leukemia cell line, thus influencing the antioxidant status [94]. According to a recent report [95], kaempferol could prevent the occurrence of hemolysis through the upregulation of antioxidant enzymes and the inhibition of ROS generation and lipid peroxidation. Moreover, it inhibited the function of phosphorylated AKT (p-AKT), cyclin D1, and cyclin-dependent kinases 4 while promoting the expression of phosphorylated breast cancer susceptibility protein, p-ATM, p53, p21, p38, Bax, and Bid, eventually evoking S phase arrest and apoptosis in bladder cancer cells. Kaempferol can also prevent carcinogenesis via modulating NRF2 signaling in MCF-10A cells [96]. Mechanistically, kaempferol upregulated the expression of NRF2 and its downstream enzyme NAD(P)H: quinone oxidoreductase 1 (NQO1), thus decreasing oxidative stress, recovering redox homeostasis, and preventing carcinogenesis. In addition, kaempferol suppressed ROS production in mouse bone marrow-derived neutrophils, which was related to the blockade of neutrophil extracellular trap formation, therefore reducing lung metastasis in a mouse breast cancer model [97]. Notably, kaempferol also decreases the ROS level by upregulating the expression of JAK/STAT3, MAPK, PI3K/AKT, and NF-κB [98,99], which are able to overcome ROS-mediated drug resistance and sensitize tumor cells to 5-fluorouracil (5-FU) [100,101]. As reported by a recent study [102], the combination of kaempferol and 5-FU blocked the production of ROS and downregulated the expression of ABC subfamily G member 2 and multidrug resistance-associated protein 1 in 5-FU-resistant LS174 cells, thus surmounting the efflux of 5-FU and drug resistance. Similarly, kaempferol could sensitize oxaliplatin (Ox)-resistant HCT116 (HCT116-OxR) cells to oxaliplatin by decreasing the transactivation activity of adaptor protein complex-1 [103]. These findings demonstrate the versatile properties of kaempferol as an antioxidant for cancer treatment.

#### 2.1.2. Resveratrol

Resveratrol, one of the most investigated polyphenols from CHMs [104,105], has been widely applied in cancer prevention due to various biological activities, especially the regulation of oxidative stress [106,107,108]. The antioxidant activities of resveratrol in cancer therapy are also related to manipulating the expression of NRF2 and its target genes, such as NQO1, SOD3, and 8-oxoguanine DNA glycosylase 1 (OGG1) [109,110], thus inhibiting estrogen-mediated DNA damage and suppressing mammary carcinogenesis [111]. In azoxymethane (AOM)- and dextran sulfate sodium (DSS)-induced cancer models, resveratrol activated crosstalk between NRF2 and mitogen-activated protein kinase phosphatase 1 to inhibit oxidative stress and prevent carcinogenesis [112]. In addition, resveratrol suppresses and abolishes the phosphorylation of key regulators, such as transforming growth factor (TGF)-β1, Smad2 and Smad3, which play essential roles in epithelial mesenchymal transition (EMT) to retard EMT and lung metastasis processes in breast cancer [113]. Moreover, resveratrol also showed the potential to overcome drug resistance. An experiment indicated that resveratrol could enhance the expression of NRF2 and suppress the level of nutrient-deprivation autophagy factor-1 (NAF-1), which led to cell death in pancreatic cancer cells [114]. More importantly, resveratrol could target NAF-1 and improve the sensitivity of pancreatic cancer cells to gemcitabine.

#### 2.1.3. Catechins

Catechins are the most abundant polyphenols in tea (the second most consumed beverage and the most commonly used CHM worldwide [115,116]), mainly including epigallocatechin-3-gallate (EGCG), epigallocatechin, epicatechin-3-gallate, and epicatechin [117,118]. Among them, EGCG is the most abundant tea polyphenol and has attracted considerable attention in cancer therapy due to its antioxidant activity [119]. Normally, EGCG can scavenge ROS and mitigate the damage of oxidative DNA and protein to protect against cancer [120]. Basically, the phenolic hydroxyl groups of EGCG endow it with the ability to combine with ROS by oxidizing the B and D ring to form stable phenoxy radicals, thereby downregulating cellular ROS levels [119]. In addition, ECCG can modulate the activity of antioxidative enzymes or oxidases to suppress the formation of byproducts such as malondialdehyde and 8-hydroxy-2′-deoxyguanosine [121,122,123]. For example, EGCG administration could inhibit the process of multistage mouse skin carcinogenesis by decreasing the expression of oxidative enzymes, such as inducible nitric oxide synthase (iNOS) and COX-2 [124]. Oral gavage of tea polyphenols, of which EGCG is the major component, could significantly prevent diethylnitrosamine/phenobarbital-induced hepatocarcinogenesis in a rat model, which was attributed to the increased total antioxidant capacity [125]. In addition, EGCG could upregulate the mRNA and protein levels of NRF2, uridine 5′-diphosphate-glucuronosyltransferase (UGT)1A, and UGT1A8, followed by the suppression of proliferation and liver and lung metastasis in an HT-29 cancer cell mouse model [126]. Similarly, oral administration of EGCG inhibited the miR483-3p-induced enhancement of human hepatocellular carcinoma cell migration and invasion by counteracting EMT markers [127]. Moreover, EGCG exhibited anticancer ability by maintaining an optimum level of NRF2 to surmount etoposide resistance in lung cancer cells by regulating the activity of KEAP1, thus inducing G2/M arrest and overcoming multidrug resistance [128].

#### 2.1.4. Other Antioxidant Polyphenols

There are many other polyphenols that can be applied in cancer treatment due to their antioxidant capability. Curcumin, a well-known polyphenol extracted from the rhizomes of Curcuma longa, was reported to inhibit the proliferation of breast cancer cells through the NRF2-mediated downregulation of Flap endonuclease 1, a DNA repair-specific nuclease. In detail, curcumin could trigger NRF2 translocation from the cytoplasm to the nucleus to bind to the promoter of Fen1 and decrease its transcriptional activity [129]. In recent studies, curcumin has been proven to increase the overall survival of cancer patients in various clinical trials [NCT01042938, NCT01160302, NCT03211104]. Wogonin, another polyphenol extracted from the root of *Scutellaria baicalensis* Georgi (a kind of CHM), has been used to treat inflammatory diseases [130]. It is now reported to promote nuclear translocation of NRF2 and counteract the levels of interleukin (IL)-6 and IL-1β, therefore relieving inflammation-associated oxidative stress and preventing colorectal carcinogenesis induced by AOM and DSS in a mouse model [131]. Moreover, quercetin, a commonly used polyphenol derived from CHMs and other edible plants, has exhibited an excellent anticancer capability by modulating redox [132]. Quercetin inhibited the migration and invasion of SAS human oral cancer cells through the downregulation of matrix metalloproteinase (MMP)-2 and MMP-9. In detail, quercetin reduced the protein activity of vascular endothelial growth factor (VEGF), NF-κB, iNOS, and COX-2 to eliminate ROS and restrain metastasis [133]. In fact, there are too many kinds of natural polyphenols to list, but they all exhibit remarkable tumor suppressing efficiency by impairing cellular oxidative stress [134,135].

**Figure 3:**
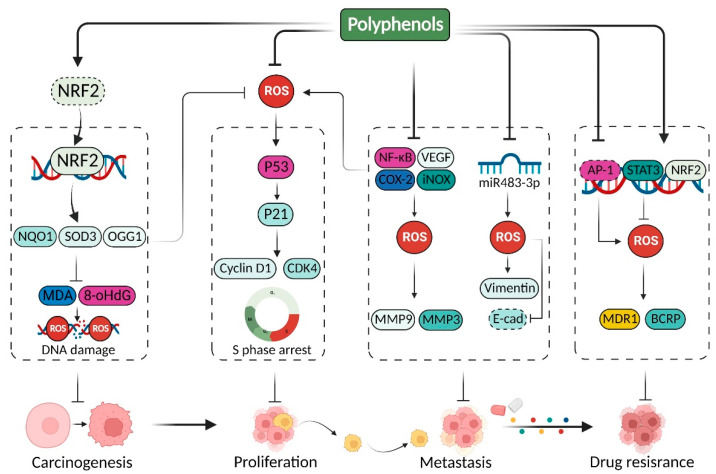
Outlines of antioxidant properties of polyphenols in cancer therapy. Polyphenols can reinforce the transcriptional activation of the classical antioxidant transcription factor NRF2 to defend against ROS-induced DNA damage, thus preventing oxidative stress-mediated carcinogenesis. Meanwhile, polyphenols could also upregulate/activate antioxidant enzymes or activate antioxidant signaling pathways to restore intracellular homeostasis for enhancing P21-mediated cell cycle arrest and blocking the epithelial mesenchymal transition (EMT) process and matrix metalloproteinase (MMP)-mediated metastasis. In addition, polyphenols are able to manipulate the activity of antioxidant or prooxidant transcription factors, thus overcoming ROS-mediated drug resistance.

Taken together, polyphenols can directly eliminate ROS to relieve oxidative stress. Additionally, they manipulate the activity of some redox signaling pathways and antioxidant enzymes to retard tumor progression, including carcinogenesis, proliferation, metastasis, and drug resistance, in a variety of cancer types (Figure 3).

### 2.2. Polyphenols Suppressive Cancer by Promoting Oxidative Stress

Several kinds of polyphenols also exhibit prooxidant capabilities in cancer therapy. Intriguingly, some kinds of polyphenols exhibit antioxidant activity, but high doses of them induce pro-oxidative stress and lead to DNA damage, lipid peroxidation, inflammasomes, and autophagosome augmentation [136,137,138,139], thus causing apoptosis, ferroptosis, autophagy, and pyroptosis in cancer cells [140,141,142,143]. Obviously, the pro-oxidant mechanism is similarly involved in the modulation of the redox signaling pathway or the activity of oxidative enzymes [144,145].

#### 2.2.1. Curcumin

A high dosage of curcumin also exhibits a pro-oxidant potential to suppress tumors. For example, curcumin could evoke cytotoxic cell death in non-small cell lung cancer (NSCLC) by promoting ROS accumulation and mitochondrial transmembrane potential reduction, thus causing DNA damage, G2/M arrest, and subsequent mitochondrial apoptosis [146]. In addition, several other ROS-mediated cell death models, such as ferroptosis, autophagy, and pyroptosis, could be induced by curcumin to kill cancer cells [147]. For instance, Tang and his coworkers revealed that curcumin administration led to iron overload, glutathione (GSH) depletion, and lipid peroxidation, causing the downregulation of Recombinant Solute Carrier Family 7, Member 11, and Glutathione peroxidase 4 (GPX4) in lung cancer cells [148]. Moreover, oxidative stress induced autolysosome accumulation and autophagy-dependent ferroptosis, consequently inhibiting the growth and proliferation of cancer cells. In hepatocellular carcinoma, curcumin intervention triggered the elevation of ROS levels to activate caspase-3 for cleavage of Gasdermin E, the executor protein of pore formation, thereby causing pyroptosis in HepG2 cells [143]. Intriguingly, curcumin could suppress the expression of key antioxidant enzymes, including SOD, CAT, GPX, and HO-1, and suspend the activity of the transcription factor NRF2, therefore increasing intracellular oxidative stress to reverse cisplatin resistance in SKOV-3 ovarian cancer cells [149]. The combined strategy of tumor necrosis factor-related apoptosis-inducing ligand (TRAIL) and curcumin use synergistically sensitized ACHN renal cancer cells to TRAIL in a mechanism of ROS-mediated activation of the JNK-CHOP pathway [150]. Similarly, its analog, L48H37, could induce ROS accumulation to evoke endoplasmic reticulum (ER) stress through phosphorylation of PERK and eIF2α and subsequently trigger cell cycle arrest and apoptosis in lung cancer cells [151].

#### 2.2.2. Wogonin

Wogonin is another compound that shows prooxidant effects in cancer treatment. For example, wogonin could elicit abundant ROS to activate caspase 3/8/9 and subsequent apoptosis, thus contributing to the inhibition of lung cancer cell proliferation [152]. In some contexts, NRF2 exerts a protective effect against chemotherapy-mediated oxidative stress, which promotes cancer cell survival and drug resistance [153]. Wogonin could downregulate the expression of NRF2 as well as its target proteins HO-1, NADP(H), and NQO-1 to impair the NRF2-mediated antioxidant system and reverse DOX resistance in MCF-7ADR breast cancer cells [154]. Further exploration demonstrated that the downregulation of NRF2 by wogonin could potentiate the cytotoxicity of chemotherapeutic agents by inhibiting multidrug resistance-associated proteins (MRPs) and inducing phage II enzymes [155]. Similarly, the inhibition of MRP1 by wogonin relies on the dissociation of NRF2 from antioxidant response elements (AREs) in human myelogenous leukemia, in which the PI3K/Akt pathway and DNA-PKcs are activated [156]. A similar mechanism was also observed in NRF2-mediated cisplatin resistance in head and neck cancer, and wogonin surmounted this phenotype [157].

#### 2.2.3. Resveratrol

Notably, resveratrol has been observed to have outstanding anticancer potential in many types of cancer cells, such as bladder, prostate, glioblastoma, colon, breast, lung, and ovarian cancer cells [158,159,160,161]. As an important source of CHM-derived polyphenols, resveratrol prevents carcinogenesis through its antioxidant ability in specific tumor types while also displaying its versatility in cancer treatment by exerting prooxidant effects. For example, a high concentration of resveratrol could induce ROS overproduction and LC3 expression and decrease mitochondrial membrane potential, which results in autophagy and proliferation suppression in HeLa cells [162]. Similarly, the increased ROS levels induced by resveratrol inhibit the activity of casein kinase 2, a protein related to proliferation and apoptosis, thus affecting mitochondrial function and the viability of breast cancer cells [163]. It has also been reported that resveratrol can inhibit the activity of antioxidant enzymes such as SOD and CAT to influence the outcome of cancer treatment. Recent studies have shown that resveratrol induces the elevation of intracellular ROS by attenuating SOD2 and CAT expression and promoting the activity of caspase-9 and caspase-3, thereby remarkably arresting proliferation and triggering extensive apoptosis in glioblastoma multiforme (GBM) cells [164]. Indeed, resveratrol could also manipulate the NRF2-related signaling pathway. In a related report, resveratrol significantly decreased the mRNA expression of Nrf2 to reverse drug resistance in HL-60ADR (Adriamycin resistant HL-60 cells) [110]. Together, these results provide a rational proposal to take advantage of the prooxidant capacity of resveratrol to combat cancer.

#### 2.2.4. Other Prooxidant Polyphenols

Some other polyphenols could also display prooxidant potential in cancer treatment. Similar to resveratrol, kaempferol can act as an oxidative stress inducer. The downregulation of NRF2 by kaempferol impaired the transcription of its target genes, including NQO-1, HO-1, AKR1C1, and GST, to enhance ROS accumulation and apoptosis in NSCLC cells [165]. Rosmarinic acid (RA), a natural polyphenol extracted from *Perilla frutescens* and *Glechoma hederacea* L. [166,167], also exhibits pro-oxidant activity. Related studies have identified that RA can trigger intracellular ROS production to upregulate the cleavage rates of caspase-8/caspase-9/caspase-3 and inhibit the expression of MMP-9/MMP-2, thus promoting apoptosis and retarding EMT in osteosarcoma cells [168]. In addition, another study reported that RA promotes ER-mediated oxidative stress to induce bax translocation and caspase-3 cleavage, which eventually triggers apoptosis in oral cancer cells [169]. Similar to RA, the prooxidant function of quercetin can be attributed to the induction of ROS-mediated oxidative stress that results from the inhibition of antioxidant enzymes or activation of oxidase [170]. For example, quercetin could induce apoptosis by decreasing mitochondrial membrane potential and inducing ER-mediated activation of activating transcription factor (ATF)-6α and ATF-6β in oral cancer SAS cells [171]. Furthermore, quercetin can increase the activity of COX-2 and elevate the intracellular ROS level, which induces apoptosis and inhibits cell survival in human colon cancer [172]. Several other studies have also demonstrated the antitumor role of quercetin by exacerbating oxidative stress and inducing cell death in a variety of tumor models [173,174].

**Figure 4:**
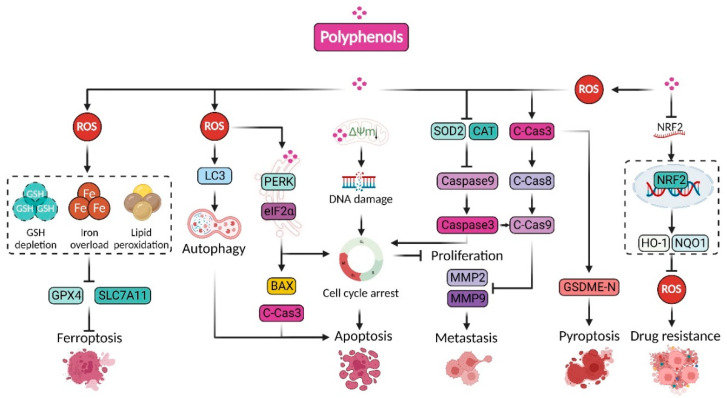
Prooxidant roles of polyphenols in cancer therapy. Typically, polyphenols induce glutathione (GSH) depletion, iron overload, lipid peroxidation, autophagosome augmentation, and caspase3 cleavage to trigger programmed death of cancer cells, including ferroptosis, autophagy, apoptosis, and pyroptosis. Moreover, polyphenols evoke endoplasmic reticulum (ER) stress and decrease in mitochondrial membrane potential, which could also induce cell death. On the other hand, polyphenol-induced ROS are capable of cleaving caspase3/8 9 and impairing the activities of MMP2/9, thus suppressing metastasis of cancer cell. In addition, polyphenols could inhibit the transcriptional activity of NRF2 to improve the sensitivity of cancer cells to chemotherapy.

Taken together, the prooxidant function of polyphenols is indispensable to manipulating redox homeostasis in cancer cells. The aberration of redox signaling and intracellular oxidative stress induced by polyphenols can lead to macromolecule damage, such as DNA or protein damage, and lipid peroxidation, which triggers programmed cell death (PCD), such as apoptosis, ferroptosis, and pyroptosis, thereby playing an essential role in cancer prevention and treatment, as well as in overcoming drug resistance (Figure 4).

## 3. Novel Strategies Facilitate the Application of Polyphenols in Cancer Therapy

**Figure 5:**
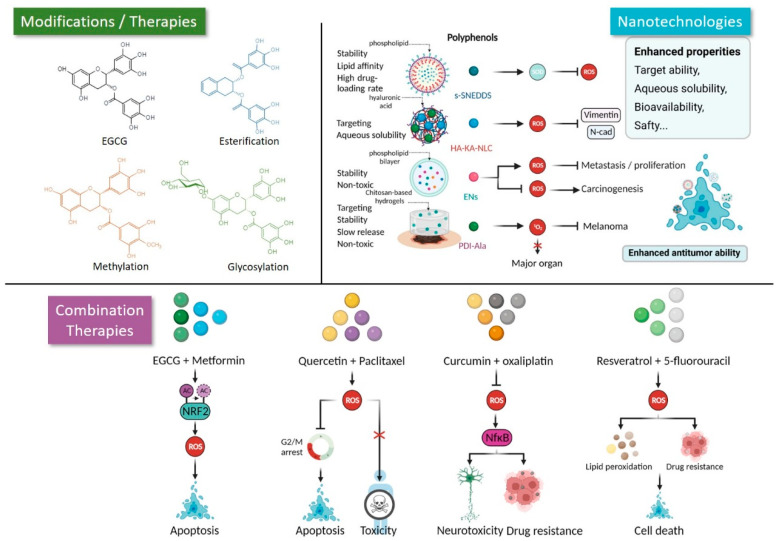
Novel strategies promote the application of polyphenols in cancer therapy. Structural modifications, such as esterification, methylation, and glycosylation, protect polyphenols from rapid degradation and promote their bioactivities. Nanotechnologies can make up the inherent limitations of polyphenols and support targeting delivery of them to specific tumor lesions with minor side effects. Combination therapies achieve efficient tumor eliminate via enhanced ROS-mediated mechanisms.

The characteristics of polyphenols in modulating redox homeostasis have been widely applied in cancer prevention and treatment, which lays the basis for the discovery and development of natural anticancer drugs. Indeed, many polyphenols have been explored in preclinical or clinical trials, but the drawbacks of polyphenols generally disturb their versatile properties in clinical settings [175]. For instance, specific structures, such as phenolic hydroxyl groups and the catechol ring of polyphenols, make them easy to oxidize [176], which greatly deteriorates their stability and increases the probability of degradation [177]. In addition, their poor water solubility, their inadequate bioavailability, and more importantly, the nonspecific selectivity of polyphenols limit their pharmacological applications [178,179]. Moreover, the single use of polyphenols always compromises their limited cytocidal effect [180]. Robust strategies have been developed to surmount these limitations to accelerate the efficient implementation of CHM-derived polyphenols for cancer treatment (Figure 5).

### 3.1. Modification

Structural modifications, such as esterification, methylation, and glycosylation, can avoid degradation and enhance the bioactivities of polyphenols. For example, the esterification of EGCG through the substitution of hydroxyl groups with the chain of fatty acids not only increased lipophilicity, but also promoted its antioxidant capacity via enhanced hydrogen atom donation [181]. Similarly, a lipophilized EGCG derivative (LEGCG) synthesized by a partial esterification reaction (an enzymatic esterification model) of EGCG with lauric acid improved its bioactivity, including anti-proliferation and pro-apoptosis effects [182]. In addition, the methylation of EGCG, which alters the phenolic hydroxyl groups of the EGCG benzene ring into methyl ether, could amend its oral absorption rate and blood stability. For instance, the in vivo bioavailability and stability of EGCG was greatly enhanced when the hydroxyl groups were replaced by more stable methoxy groups [183]. Similarly, the bioavailability of methylated EGCG was higher than that of free or unmethylated EGCG [184]. The glycosylation of polyphenols can improve their solubility and stability and protect these compounds from oxidants, light degradation, and hostile gastrointestinal conditions [185,186]. Intriguingly, the glycosylated EGCG could act as a prodrug and first be deglycosylated at the intestinal surface before diffusing into enterocytes, thereby increasing the stability of EGCG during processing, storage, and gut transit after ingestion [187]. Moreover, the modifications for polyphenols could enhance the purification efficiency, prolong the preservation time, and avoid degradation in elevated large-scale production and commercialization [188,189,190]. Taken together, the modification of polyphenols evades rapid degradation and enhances their accessibility, thus providing rational strategies to accelerate the application of polyphenols in cancer treatment.

### 3.2. Nano Strategies

Recent evidence has shown that nano strategies can aid in overcoming polyphenols’ inherent drawbacks, including their low water solubility, poor stability, and nontargeting ability [191,192]. A myriad of nano strategies has been well established for improving pharmacokinetic properties under polyphenol administration [193,194]. Some nanocarriers, such as nanoparticles, liposomes, hydrogels, and extracellular vehicles, are widely applied to deliver polyphenols for cancer treatment [195,196,197]. The nanostructured lipid carrier can encapsulate kaempferol to optimize its low aqueous solubility and poor bioavailability [198]. After being modified with hyaluronic acid (HA), the HA-KA-NLC nanoplatform could target NSCLC cells by recognizing highly expressed CD44, thus exhibiting more efficient inhibition of their proliferation and EMT than free kaempferol administration. Indeed, these nano strategies strengthened redox regulation, which synergizes with the enhanced bioavailability to greatly improve therapeutic efficiency. One recent study reported a solid self-nanoemulsifying drug delivery system (s-SNEDDS) loaded with resveratrol and tamoxifen to treat breast cancer [199]. The s-SNEDDS team not only improved the bioavailability of resveratrol, but also sensitized tamoxifen-mediated chemotherapy and exhibited satisfactory suppression of MCF-7 breast cancer cells by triggering resveratrol-induced ROS elimination and SOD activation. In addition, a tannic acid-loaded dual antioxidant-photosensitizing hydrogel system was established to protect against human melanoma. In this nanosystem, chitosan-based hydrogels were designed using tannic acid as an antioxidant cross-linker and loaded with photosensitizer PDI-Ala, in which the tannic acid controlled the ROS generation and minimized the side effects of singlet oxygen synergistically with PDI-Ala boosted photodynamic therapy [200]. Recently, natural exosome-like nanovesicles (ENs) have endured much investigation as novel carriers and therapeutic agents. In Zu’s study, ENs extracted from green tea leaves were rich in polyphenols such as EGCG, quercetin, and myricetin and exhibited distinct efficiency in preventing AOM- and DSS-induced colorectal carcinogenesis in a mouse model by maintaining intracellular redox homeostasis [201]. Similarly, tea flower-derived ENs could inhibit breast cancer metastasis by stimulating ROS amplification [202]. Taken together, nano strategies ideally tackle the limitations of polyphenols, making them promising agents for cancer treatment.

### 3.3. Combination with Other Agents

Strategies to combine polyphenols with other agents play important roles in preclinical evaluation and clinical implementation, including amplifying ROS-mediated therapeutic efficiency and avoiding chemotherapy-induced side effects [203]. The combinational use of EGCG with metformin, a classical antidiabetic drug, stimulated intracellular ROS accumulation induced by EGCG (100 μM) through the modulation of Sirtuin 1-dependent deacetylation on NRF2, thus augmenting the anticancer effect of EGCG in NSCLC treatment [204]. Indeed, the combination of polyphenols with first-line chemotherapies such as paclitaxel, 5-fluorouracil, and oxaliplatin could not only augment the cytocidal function but also reduce the side effects of these chemotherapies [205,206]. In addition, combined treatment with quercetin and paclitaxel could significantly inhibit proliferation and migration and evoke apoptosis via increased ROS generation, as well as attenuate the side effects of paclitaxel in PC-3 prostate cancer cells [207]. Similarly, the combination of curcumin also sensitizes cancer cells to oxaliplatin and alleviates oxaliplatin-induced peripheral neuropathic pain by inhibiting the oxidative stress-mediated activation of NF-κB [208,209,210]. Interestingly, a recent phase I trial showed the safety, tolerability, and feasibility of administering curcumin as an adjunct to FOLFOX (5-fluorouracil, folinic acid, and oxaliplatin) chemotherapy in patient-derived colorectal or liver metastases cancer [NCT01490996]. Alternatively, resveratrol exhibited a synergetic effect with 5-fluorouracil to induce an imbalance in cellular antioxidant activities and subsequent intracellular ROS accumulation and lipid peroxides, thus leading to a significant decrease in long-term colon cell survival [211]. In summary, these novel strategies ingeniously alleviate the predicament of polyphenols and enhance the feasibility of developing new agents with redox regulation’s ability to overcome cancer progression.

## 4. Conclusions and Perspective

Oxidative stress acts as a double-edged sword in tumor progression; thus, the strategy to manipulate ROS levels to influence redox homeostasis is significant for targeting tumors. Notably, polyphenols can regulate oxidative stress in cancer treatment. In this review, focusing on redox homeostasis modulation and the involved molecular mechanisms, we have summarized several polyphenols that can function at different stages of cancer progression. We have also discussed several novel strategies, including chemical modification, nanotechnology, and combined treatment, that polyphenols could facilitate as optimum therapeutic agents in clinical settings.

A myriad of preclinical and clinical trials has demonstrated the potential value of polyphenols in the prevention of carcinogenesis, limitation of proliferation, inhibition of metastasis, and overcoming of drug resistance. Given that a large number of polyphenols are derived from CHMs and are easily acquired from a common daily diet, polyphenols are recognized as promising candidates for cancer therapy. However, several issues remain to be resolved before they can actually be used in the clinic. Primarily, their poor solvability, targeting ability, and stability seriously impede their application. In addition, some reports revealed that several antioxidants lead to the progression of tumors [212,213,214]. Moreover, the precision of dosage and pharmacokinetics remains a realistic problem in gastrointestinal transit through oral administration [215]. For these considerations, several strategies, including chemical modification, nanotechnology, and combined treatment, have been adopted to optimize the project in daily application. It is worth noting that the antagonistic potential of polyphenols reminds us to establish rigorous in vitro and in vivo screening systems before creating daily use and depth methods to explore the accuracy of dosage and pharmacokinetics. Furthermore, novel strategies still need investment. Favorable clinical data are also largely needed to confirm their tangible benefit in cancer therapy. Only through accruing such knowledge and developing these strategies will polyphenols become an optimum therapeutic agent in daily application.

## Data Availability

Not applicable.
